# COVID-19 Testing Unit Munich: Impact of Public Health and Safety Measures on Patient Characteristics and Test Results, January to September 2020

**DOI:** 10.3389/fpubh.2022.856189

**Published:** 2022-03-22

**Authors:** Hannah Tuulikki Hohl, Christian Heumann, Camilla Rothe, Michael Hoelscher, Christian Janke, Guenter Froeschl

**Affiliations:** ^1^Division of Infectious Diseases and Tropical Medicine, University Hospital, LMU Munich, Munich, Germany; ^2^Department of Statistics, Faculty of Mathematics, Informatics and Statistics, University of Munich, Ludwig-Maximilians-Universität (LMU), Munich, Germany; ^3^German Center for Infection Research (DZIF), Partner Site Munich, Braunschweig, Germany

**Keywords:** SARS-CoV-2, COVID-19, testing unit, public health, epidemiology, Munich, Germany

## Abstract

To assess the course of the COVID-19 pandemic and the impact of non-pharmaceutical interventions, the number of reported positive test results is frequently used as an estimate of the true number of population-wide infections. We conducted a retrospective observational analysis of patient data of the Corona Testing Unit (CTU) in Munich, Bavaria, Germany between January 27th, and September 30th, 2020. We analyzed the course of daily patient numbers over time by fitting a negative binomial model with multiple breakpoints. Additionally, we investigated possible influencing factors on patient numbers and characteristics by literature review of policy papers and key informant interviews with individuals involved in the set-up of the CTU. The 3,963 patients included were mostly young (median age: 34, interquartile range: 27–48), female (66.2%), and working in the healthcare sector (77%). For these, 5,314 real-time RT-PCR tests were conducted with 157 (2.94%) positive results. The overall curve of daily tests and positive results fits the re-ported state-wide incidence in large parts but shows multiple breakpoints with considerable trend changes. These can be most fittingly attributed to testing capacities and -strategies and individual risk behavior, rather than public health measures. With the large impact on patient numbers and pre-test probabilities of various strategic and operational factors, we consider the derived re-ported incidence as a poor measurement to base policy decisions on. Testing units should be prepared to encounter these fluctuations with a quickly adaptable structure.

## Introduction

Since the outbreak of SARS-CoV-2 in autumn 2019 and its subsequent spread worldwide, the pandemic has caused more than 5.2 million deaths worldwide (1). Countries have implemented public health and safety measures (PHSM) to confront the spread of the virus, including social distancing measures, face masks, quarantine, and different testing strategies. The effect of these PHSM on the course of the pandemic has been studied extensively (2, 3). Moreover, numerous epidemiological models for the prediction of infection rates over time and the effects of different PHSM have been developed (4, 5). To estimate the true number of infections, population-based studies frequently utilize the number of reported positive test results, as do the World Health Organization and the European Centre for Disease Control in their epidemiological updates (6, 7). At the same time, emerging evidence suggests the re-ported incidence alone to poorly depict the course of the pandemic (8, 9). As of 31 August 2021, the German Parliament has announced to move away from the 7-day incidence as the central benchmark for imposing protective measures (10). We aim to investigate the factors influencing patient characteristics and test results on the example of the Corona Testing Unit Munich (CTU) to demonstrate the context in which this data is generated and to gain understanding on the validity of the number of positive tests as an estimate of true incidence in a population. Additionally, we aim for our findings to potentially serve other professionals with the organization of similar testing units in the future.

## Materials and Methods

### Study Setting

The Corona Testing Unit Munich was instituted by the outpatient department of the Division of Infectious and Tropical Medicine of the University Hospital of the Ludwig-Maximilians-Universität Munich (LMU) in the state of Bavaria, Germany, after testing the first positive patient in Germany at the same outpatient department on January 27th, 2020 (11).

Until its closing in March 2021, the CTU performed over 10.000 SARS-CoV-2 tests in the first and second wave of the Covid-19 pandemic in Bavaria (12).

### Study Design

We conducted a retrospective analysis of patient data collected as part of the clinical practice between January 27th and September 30th, 2020. To complement the analysis and interpret the results, we reviewed policy papers and regulations enacted in Bavaria in the given timeframe. Additionally, we conducted key informant interviews with stakeholders actively involved in the set-up of the CTU.

We chose an interpretative approach (that combined quantitative and qualitative aspects of data collection) to provide a broader perspective on the research topic. The study is reported according to STROBE guidelines for observational cohort studies in epidemiology.

### Statistical Analysis of Patient Data

#### Inclusion Criteria and Collected Data

Between January 27th and September 30th, 2020, 3,983 patients and asymptomatic clients (both subsequently collectively referred to as patients) were admitted to the CTU for SARS-CoV-2 testing. All patients with responding case report forms (CRF) or equivalent data saved in the patient records were included.

Patients were admitted through self-referral or referral by cooperating institutions. In case of self-referral, the triage and decision for testing was up to the discretion of our clinicians and was carried out in accordance with current guidelines by the Robert Koch Institute (RKI), a German federal government agency and research institute responsible for disease control and prevention. Triage criteria included a recent stay in a risk area (as designated by RKI), contact to a confirmed COVID-19 case and COVID-specific symptoms. From September 7th, 2020 on, the CTU was open for the general public for indication-free testing through the Bayerische Teststrategie (BTS), a state-wide testing program.

Anamnesis was initially taken by our team of physicians in a common patient interview. From February 27th on, we used a structured CRF, which then patients were asked to fill out themselves. Questions on the CRF included sociodemographic information, possible expositions, and symptoms (see Supplementary File 1).

Naso- or oropharyngeal swabs were carried out by a team of physicians and trained medical students (13). Laboratory analysis of the samples was carried out by the Institute for Microbiology of the Armed Forces in Munich, the Max-von-Pettenkofer-Institute of the Ludwig-Maximilians-Universität Munich and the private medical laboratory Labor Becker & Kollegen in Munich. Covid-19 was confirmed using real time quantitative reverse transcription polymerase chain reaction (qRT-PCR).

#### Data Entry

We entered the data derived from case report forms and patient records into an Excel 2010 database. Free text information was adopted verbatim. Not readable or in-consistent information noted by patients was reviewed by a second research team member and then labeled as missing data. Questions with pre-defined multiple-choice fields included gender, specifics to any close contact with confirmed COVID-cases and a symptom check list. Early CRF versions (before March 23rd) included additional selectable details for close contacts with COVID-cases, which are grouped under “other” for this analysis.

#### Data Analysis

We used frequencies for nominal and medians and interquartile ranges for ordinal categorical variables to represent patient characteristics, possible exposures, and testing outcomes. In case of patients with multiple tests over time, each testing occasion was analyzed independently, unless specified otherwise.

We grouped patients by their main admission motive: The group “Webasto” includes all patients with ties to the first COVID-outbreak in Germany, which took place in the car part manufacturing company Webasto, as previously described (11). “Returning Travelers” are all patients with a self-reported, recent travel history. We defined Healthcare Workers (HCW) as staff of healthcare providers, mostly employees of hospitals and nursing homes referred to the CTU by their company doctors, and self-referred patients with a profession in the health sector. One collaborating hospital referred pre-surgical intervention patients to the CTU for SARS-CoV-2-clearance, which are put in the category “pre-OP patients.” Additionally, we assisted during a COVID-outbreak in a secondary school in Munich. These patients are grouped as “School Cohort.” All other patients are put in the category “Others.” In case of coincidence with multiple groups, the main admission motive was estimated.

To differentiate between strong- and light- or asymptomatic patients, we defined strong symptoms as two or more symptoms and/or one of the following: fever, chest pain, wheezing, confusion, seizure. For a sub-analysis of the number of days between symptom onset and testing date, we excluded 118 patients with a calculated timeframe under 0 and over 14 days, the former being contradictory and the latter presumably indicating the symptoms not to be the primary reason for presentation.

Furthermore, we visually analyzed the travel patterns of patients in the context of risk areas announced by the RKI over time (14) with map coordinates by Natural Earth (15).

To analyze the temporal course of patient numbers and identify trend changes, we adopted a regression model with multiple breakpoints for patient numbers per 7 day moving average (16). We chose log-normal and negative binomial models as the most closely fitting to our dataset. Considering the rather limited number of data points we excluded models with more than eight change points to avoid overfitting. The optimal number of breakpoints was determined by lowest Bayesian Information Criterion (BIC). We then compared our log normal and negative binomial model by their respective *R*^2^-values. As the negative binomial model showed better performance, it was selected for final analysis.

General analysis was performed with STATA (version 16.1). Breakpoint analysis was conducted with package “segmented” (version 1.3-4), R (version 4.0.4).

### Context Information: Key Informant Interviews and Gray Literature

#### Key Informant Interviews

We used purposive sampling to identify individuals who were directly involved in the set-up and organization of the testing unit. The interview participants included a physician and principal investigator of the CORESA study, a study on outbreak dynamics in retirement homes in Munich; the head of CTU; and the head of outpatient department of the institute. Due to the focus of the key informant interviews on retrospectively exploring the sequence of events rather than development of a new theoretical framework solely based on the interviews, data saturation could be reached after three interviews.

We used a semi-structured interview guide to gain perspectives about influencing factors and dates potentially relevant to interpreting patient numbers. Interviews were taken by one team member (HTH) in person or over online video call between April 12th and June 9th, 2021 in English and lasted between 1 h 30 min and 2 h. The interviews were audio recorded and additionally, interview notes were taken.

#### Literature Review

Additionally, we performed a scoping review of gray literature regarding public health and safety measures in Bavaria, Germany (17, 18), as well as internal team logs and communications. Official gazettes, reports and press announcements of the Bavarian State Government, the Council of Ministers and the Crisis Committee of the German Federal Ministry of Health were searched for recommendations, regulations and laws concerning the COVID-19 pandemic (19–22). We searched the Website of the RKI for public health and safety recommendations concerning the COVID-19 pandemic, including the appointment of international risk areas (23).

#### Timeline

To visualize possible influences on patient numbers and characteristics we grouped the aspects ascertained through key informant interviews and literature research considered most relevant by our research team thematically on separate timelines.

## Results

### General Patient Characteristics by Age Group

In the observed time period, 3,963 patients were attended at the CTU in which 5,314 SARS-CoV-2 tests were conducted (see Table 1). The majority were female (66.2%) and the median age was 34 [interquartile range (IQR): 27–48]. SARS-CoV-2 PCR was negative in 5,167 (96.78%) and positive in 157 (2.94%) cases; 15 (0.28%) tests achieved no viable result.

**Table 1:** Patient characteristics: frequency and percentage by age group and test result.

**Age groups**	**0–19 years**				**20–39 years**				**40–59 years**				**60–89 years**				**Total**			
**Characteristics**	**Negative**		**Positive**		**Negative**		**Positive**		**Negative**		**Positive**		**Negative**		**Positive**		**Negative**		**Positive**	
	**(*****n*** **= 380)**		**(*****n*** **= 4)**		**(*****n*** **= 2,762)**		**(*****n*** **= 80)**		**(*****n*** **= 1,725)**		**(*****n*** **= 65)**		**(*****n*** **= 298)**		**(*****n*** **= 8)**		**(*****n*** **= 5,167)**		**(*****n*** **= 157)**	
**Gender**																				
Female	200	52.6%	2	50.0%	1,779	64.4%	54	67.5%	1,245	72.2%	45	69.2%	194	65.1%	5	62.5%	3,420	66.2%	106	67.5%
Male	180	47.4%	2	50.0%	983	35.6%	26	32.5%	480	27.8%	20	30.8%	104	34.9%	3	37.5%	1,747	33.8%	51	32.5%
**Patient Group**																				
Webasto cohort	–		–		6	0.2%	2	2.5%	5	0.3%	2	3.1%	–		–		11	0.2%	4	2.5%
Travel returnees	36	9.5%	2	50.0%	281	10.2%	8	10.0%	154	8.9%	6	9.2%	23	7.7%	0	0.0%	495	9.6%	16	10.2%
HCW	76	20.0%	2	50.0%	2,270	82.2%	63	78.8%	1,453	84.2%	53	81.5%	201	67.4%	8	100.0%	4,001	77.4%	126	80.3%
Pre-OP patients	1	0.3%	0	0.0%	28	1.0%	1	1.3%	24	1.4%	0	0.0%	50	16.8%	0	0.0%	103	2.0%	1	0.6%
School cohort	164	43.2%	0	0.0%	7	0.3%	0	0.0%	13	0.8%	0	0.0%					184	3.6%	0	0.0%
Others	103	27.1%	0	0.0%	170	6.2%	6	7.5%	76	4.4%	4	6.2%	24	8.1%	0	0.0%	373	7.2%	10	6.4%
**Occupation**																				
Caregiver	–		–		–		–		19	1.1%	0	0.0%	8	2.7%	0	0.0%	27	0.5%	0	0.0%
Administration	–		–		19	0.7%	0	0.0%	37	2.1%	1	1.5%	5	1.7%	0	0.0%	61	1.2%	1	0.6%
Cleaner	–		–		15	0.5%	0	0.0%	15	0.9%	2	3.1%	2	0.7%	0	0.0%	32	0.6%	2	1.3%
Employee	2	0.5%	0	0.0%	19	0.7%	0	0.0%	11	0.6%	0	0.0%	–		–		32	0.6%	0	0.0%
Occupational therapist	–		–		49	1.8%	0	0.0%	8	0.5%	0	0.0%	–		–		57	1.1%	0	0.0%
Housekeeping	–		–		14	0.5%	0	0.0%	15	0.9%	0	0.0%	2	0.7%	0	0.0%	31	0.6%	0	0.0%
No information	160	42.1%	4	100.0%	1,604	58.1%	54	67.5%	1,033	59.9%	46	70.8%	189	63.4%	5	62.5%	2,988	57.8%	109	69.4%
Nurse/Geriatric nurse/Nursing assistant	12	3.2%	0	0.0%	451	16.3%	15	18.8%	252	14.6%	8	12.3%	33	11.1%	3	37.5%	748	14.5%	26	16.6%
Other occupation	7	1.8%	0	0.0%	230	8.3%	1	1.3%	222	12.9%	3	4.6%	43	14.4%	0	0.0%	502	9.7%	4	2.5%
Physician	1	0.3%	0	0.0%	178	6.4%	6	7.5%	62	3.6%	2	3.1%	8	2.7%	0	0.0%	249	4.8%	8	5.1%
Physiotherapist	–		–		68	2.5%	2	2.5%	19	1.1%	2	3.1%	–		–		87	1.7%	4	2.5%
Speech therapist	–		–		29	1.0%	0	0.0%	9	0.5%	0	0.0%	1	0.3%	0	0.0%	39	0.8%	0	0.0%
Student	192	50.5%	0	0.0%	56	2.0%	2	2.5%	6	0.3%	0	0.0%	1	0.3%	0	0.0%	255	4.9%	2	1.3%
Teacher	1	0.3%	0	0.0%	12	0.4%	0	0.0%	15	0.9%	0	0.0%	1	0.3%	0	0.0%	29	0.6%	0	0.0%
Trainee	3	0.8%	0	0.0%	17	0.6%	0	0.0%	1	0.1%	1	1.5%	–		–		21	0.4%	1	0.6%
Unemployed	2	0.5%	0	0.0%	1	0.0%	0	0.0%	1	0.1%	0	0.0%	5	1.7%	0	0.0%	9	0.2%	0	0.0%
**Symptoms**																				
Strong symptoms	86	22.6%	4	100.0%	740	26.8%	39	48.8%	437	25.3%	39	60.0%	60	20.1%	7	87.5%	1,325	25.6%	89	56.7%
Light/No symptoms	291	76.6%	0	0.0%	1,947	70.5%	38	47.5%	1,258	72.9%	25	38.5%	228	76.5%	1	12.5%	3,724	72.1%	64	40.8%
No information	3	0.8%	0	0.0%	75	2.7%	3	3.8%	30	1.7%	1	1.5%	10	3.4%	0	0.0%	118	2.3%	4	2.5%
Days since symptom onset^*^	5.2	3.0	6.0	7.0	5.4	5.0	4.6	3.0	5.7	5.0	4.6	4.0	11.4	8.5	3.8	2.0	5.7	5.0	4.6	3.0
**Close Contact to Positive Case**																				
Colleague	25	7.4%	2	50.0%	828	32.9%	30	41.1%	607	40.4%	21	36.2%	88	37.9%	4	57.1%	1,548	33.7%	57	40.1%
Patient	13	3.8%	2	50.0%	590	23.4%	27	37.0%	332	22.1%	19	32.8%	42	18.0%	2	28.6%	977	21.3%	50	35.2%
Private	58	16.7%	0	0.0%	204	8.0%	8	10.7%	111	7.3%	8	13.6%	21	8.9%	0	0.0%	395	8.5%	16	11.0%
Other exposition	112	32.3%	0	0.0%	178	7.0%	8	10.7%	87	5.7%	2	3.4%	13	5.5%	3	42.9%	390	8.4%	13	9.0%
No exposition	104	30.0%	1	25.0%	837	32.7%	9	12.0%	448	29.6%	12	20.3%	90	38.3%	0	0.0%	1,480	31.8%	22	15.2%
No information	43	11.3%	1	25.0%	258	9.3%	4	5.0%	243	14.1%	7	10.8%	67	22.5%	1	12.5%	611	11.8%	13	8.3%

* *Days Since Symptom Onset: Mean and Inter-Quartile Range*.

Of the observed patient groups, HCW (77%) was the overall predominant category, although in age group 0–19 years, 42.7% were affiliated with the School Cohort and 16.8% of the 60- to 89-year-olds presented for pre-surgical testing. Of the 41.8% of patients who provided information on their profession, healthcare related occupations were the most frequent, with nursing professionals comprising 14.5% of all patients. 50.0% of the under 20-year-olds stated to be students.

In 56.7% of all tests with SARS-CoV-2-positive result patients presented with strong symptoms, compared to 25.6% of negative tests. Although a substantial dispersion must be noted, the mean time between symptom onset and presentation can be observed to be higher in negative compared to positive patients (5.7 vs. 4.6 days) and to rise with patient age.

The overall most frequently reported exposition to a confirmed COVID-19-case was to colleagues (33.9%), followed by patients (21.7%), private contacts (8.6%), and “other contacts” (8.4%). 31.8% of negative and 15.2% of positive patients reported not to have had any close contact with a SARS-CoV-2 positive individual.

### Trends in Patient Numbers Over Time

**Figure 1:**
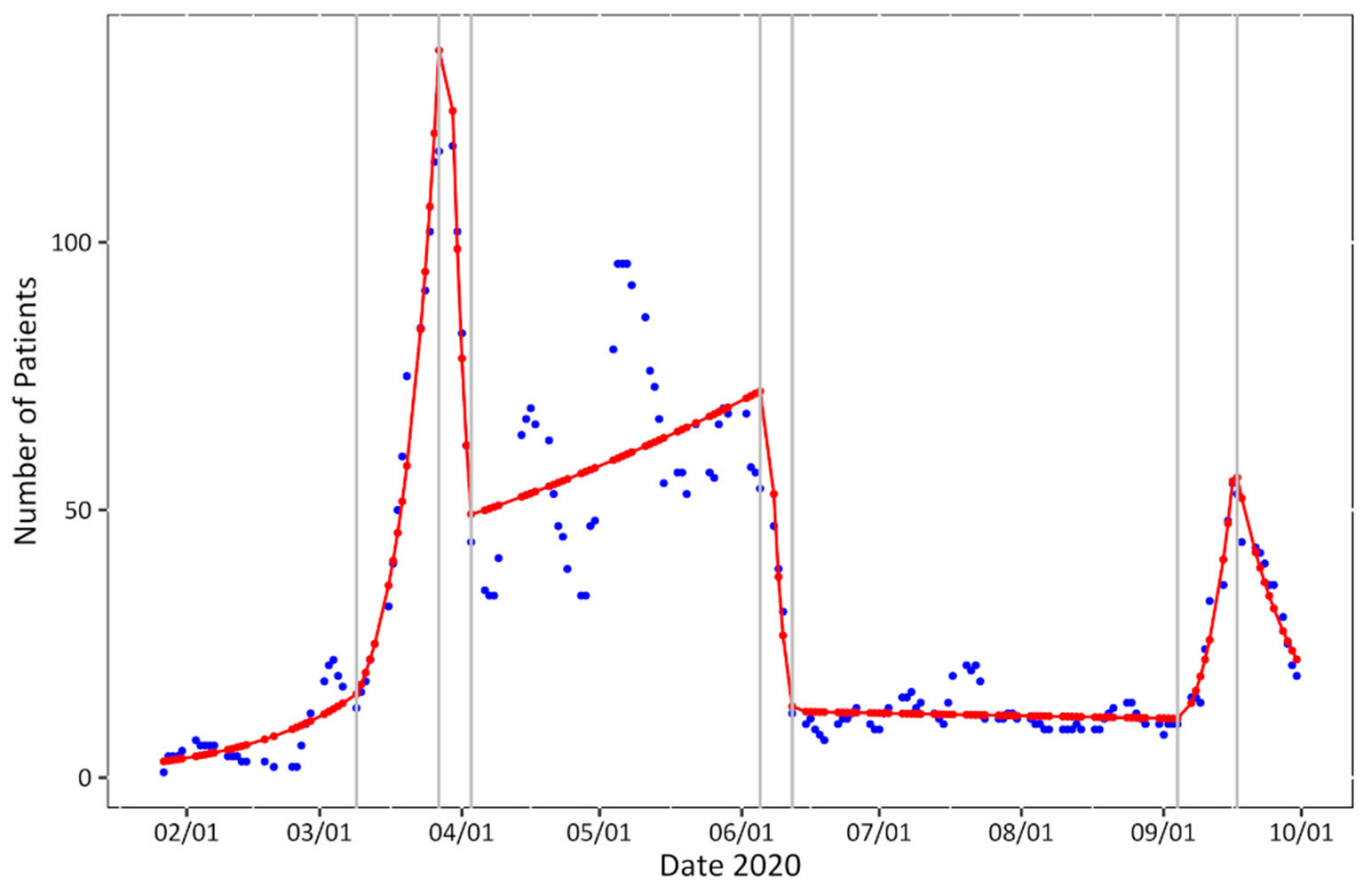
Patient numbers over time. Seven-day moving average of daily patient numbers (blue), negative binomial model (red) with breakpoints (gray).

Although Figure 1 represents the moving average and therefore a naturally rounded depiction of daily patient numbers, highly fluctuating numbers with several abrupt trend changes can be observed. The course of the readings can be most fittingly described with a negative binomial model with seven breakpoints on March 9th, March 28th, April 3rd, June 7th, June 12th, September 5th, and September 16th (BIC = 1159.645, *R*^2^ = 0.9506854).

### Trends in Patient Characteristics Over Time

We observed distinct changes in all studied categories of patient characteristics over time.

#### Patient Groups

**Figure 2:**
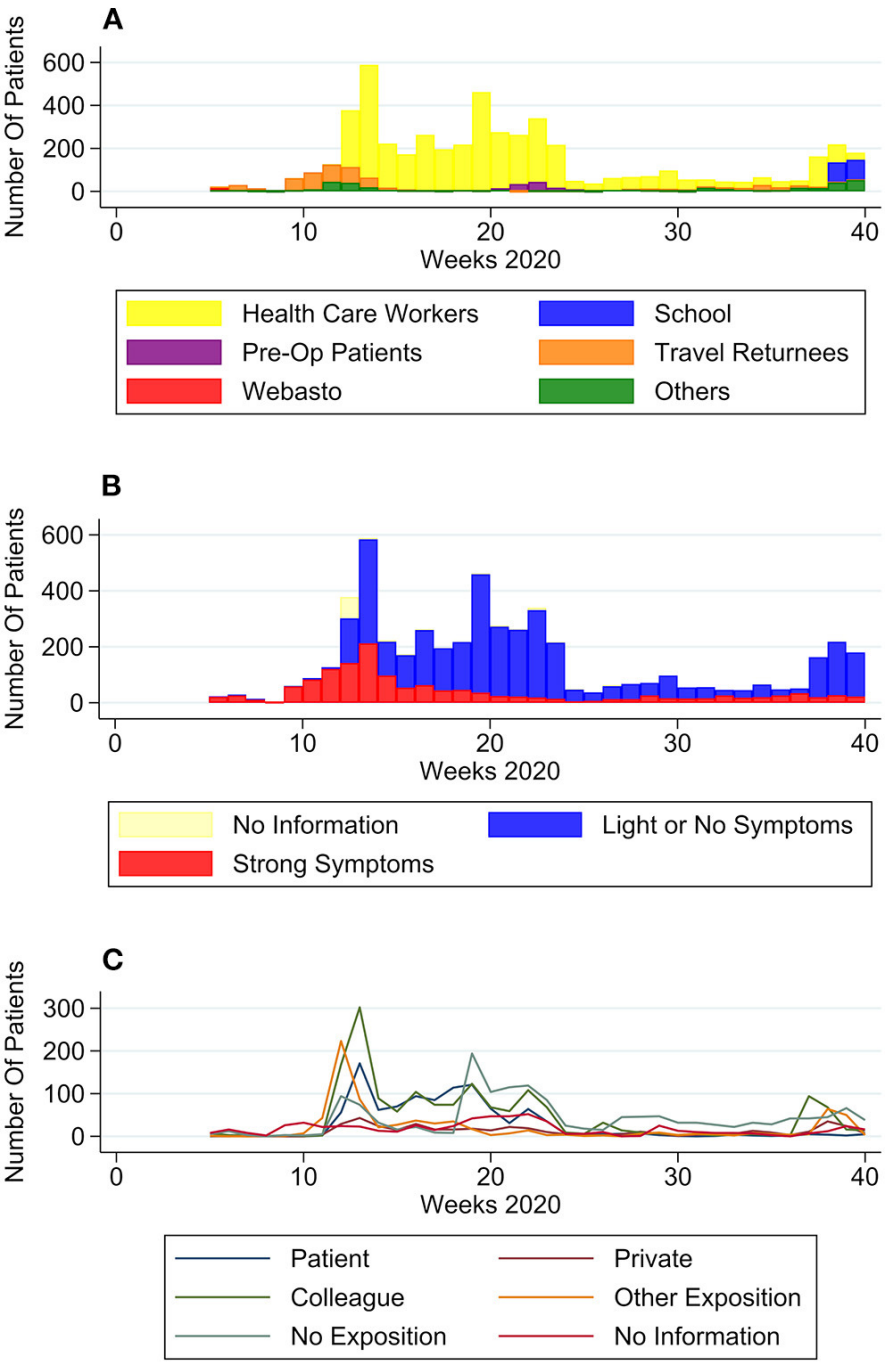
Self-reported patient characteristics over time. **(A)** Number of patients per group over time, **(B)** number of patients with no to light symptoms or strong symptoms over time, and **(C)** number of patients per type of exposition to COVID-19 positive case.

Some groups showed only temporary rises in numbers over 2–5 weeks (the Webasto Cohort, Pre-Op patients and the School Cohort) whereas others (Travel Returnees, HCW) showed elevated numbers over longer time (Figure 2A). Until the end of week 11, the majority of patients reported to be travel returnees. This group sees another slight rise between week 31 and 37, although far less pronounced. From week 12 on, HCW are by far the predominant group. Patients without group affiliation have their highest numbers in week 11 and 40.

#### Symptoms

**Figure 3:**
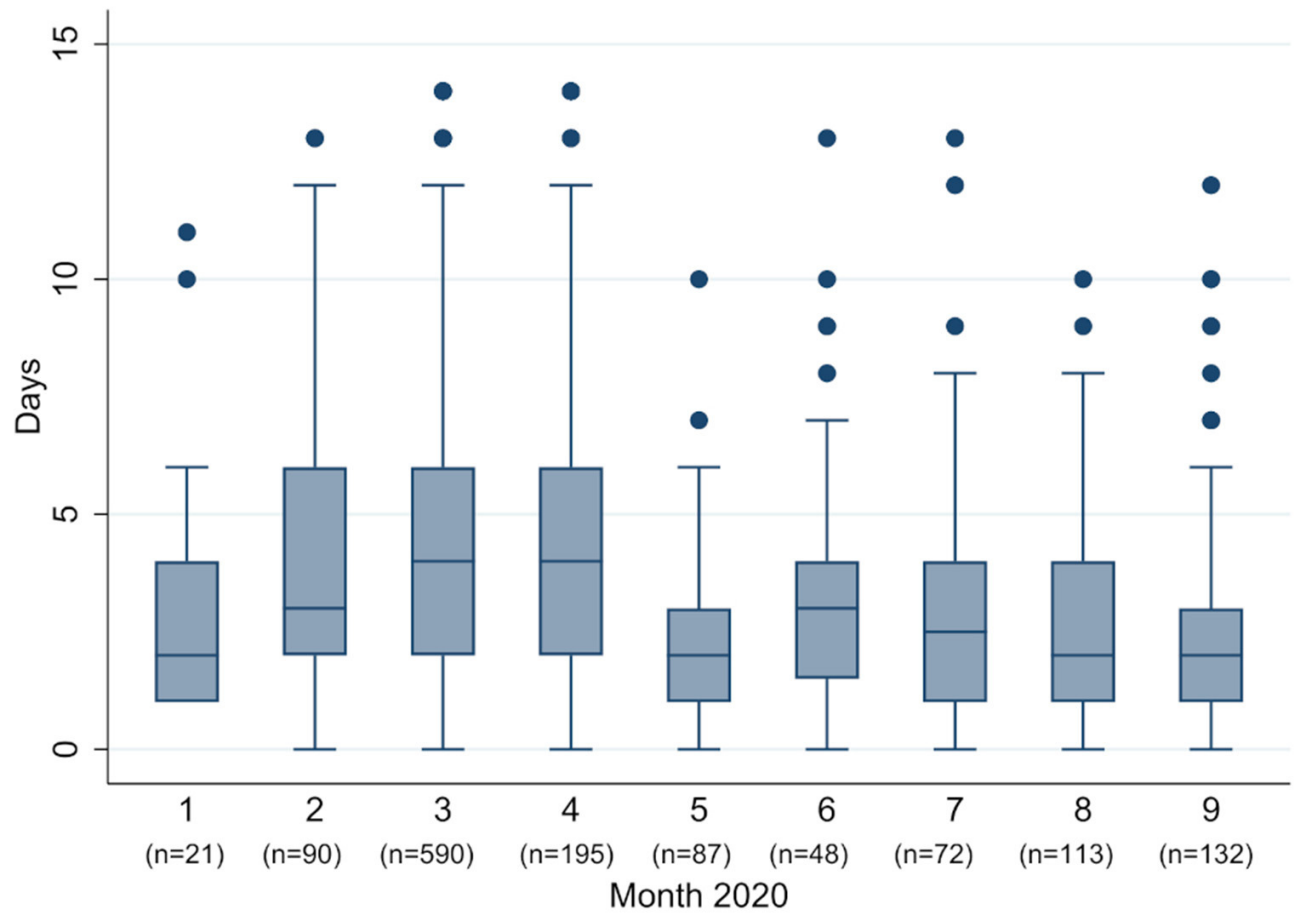
Box plot of days since onset of symptoms, per month.

In January and February (week 5–11) most patients (93.9%) presented with strong symptoms (Figure 2B). Numbers of patients with strong symptoms continued to rise, until falling after week 14 and staying to a consistent low. Week 37 saw a significant rise in percentage of patients with light or no symptoms (from 33.3% in week 36–87.7% in week 37). Although symptomatic patients reported a big range of days since symptom onset, nevertheless a trend in numbers can be observed (Figure 3): From February to April the median moved within the frame of 4–5 days, whereafter a break in trend can be seen, with a median of 2–4 days from May to September.

#### Expositions

Figure 2C shows changes in contacts with SARS-CoV-2-positive individuals:

The number of patients with close contact to a positive patient at their workplace had its' peak in April, with a subsequent decline. Numbers for exposition to a positive private contact, although comparatively low, can be seen to rise over the observed time period. Positive colleagues were overall the most frequently stated contact group, except a dip in week 24–36.

**Figure 4:**
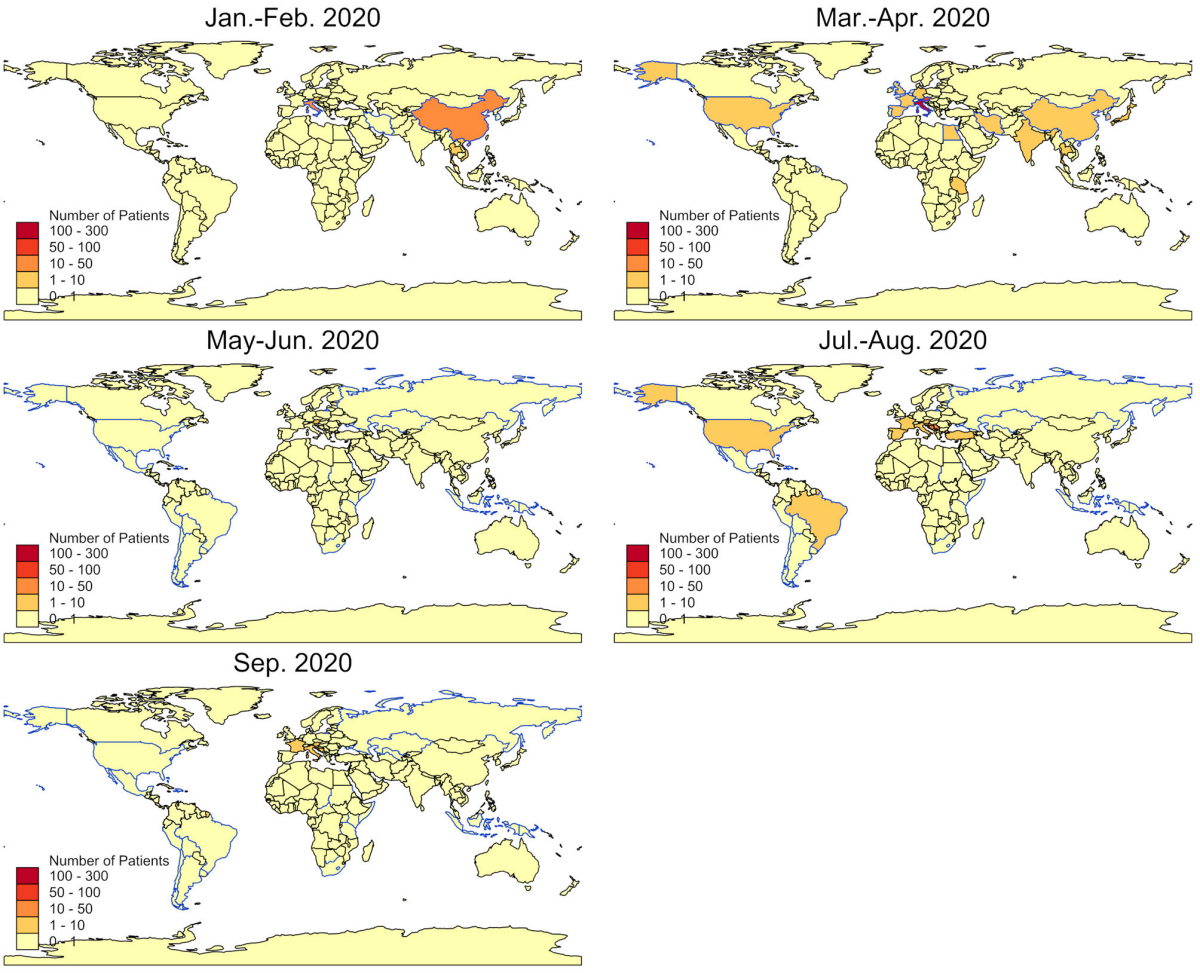
Countries of reported travel history. Bimonthly numbers of travel returnees admitted to the CTU by country (colored areas) and risk countries as by RKI (blue outlines).

Two periods of time with increased travel volume can be observed (Figure 2A): From January to mid-April (week 5–15), 387 patients reported recent travels abroad, with the most frequently travel destinations including Austria (*n* = 79), China (*n* = 42), and Italy (*n* = 219; Figure 4). From week 16 to 40, 126 patients specified recent travel destinations, the three most frequently stated being Bosnia and Herzegovina (*n* = 36), Croatia (*n* = 18), and Serbia (*n* = 13).

#### Timeline of Possible Influencing Factors

**Figure 5:**
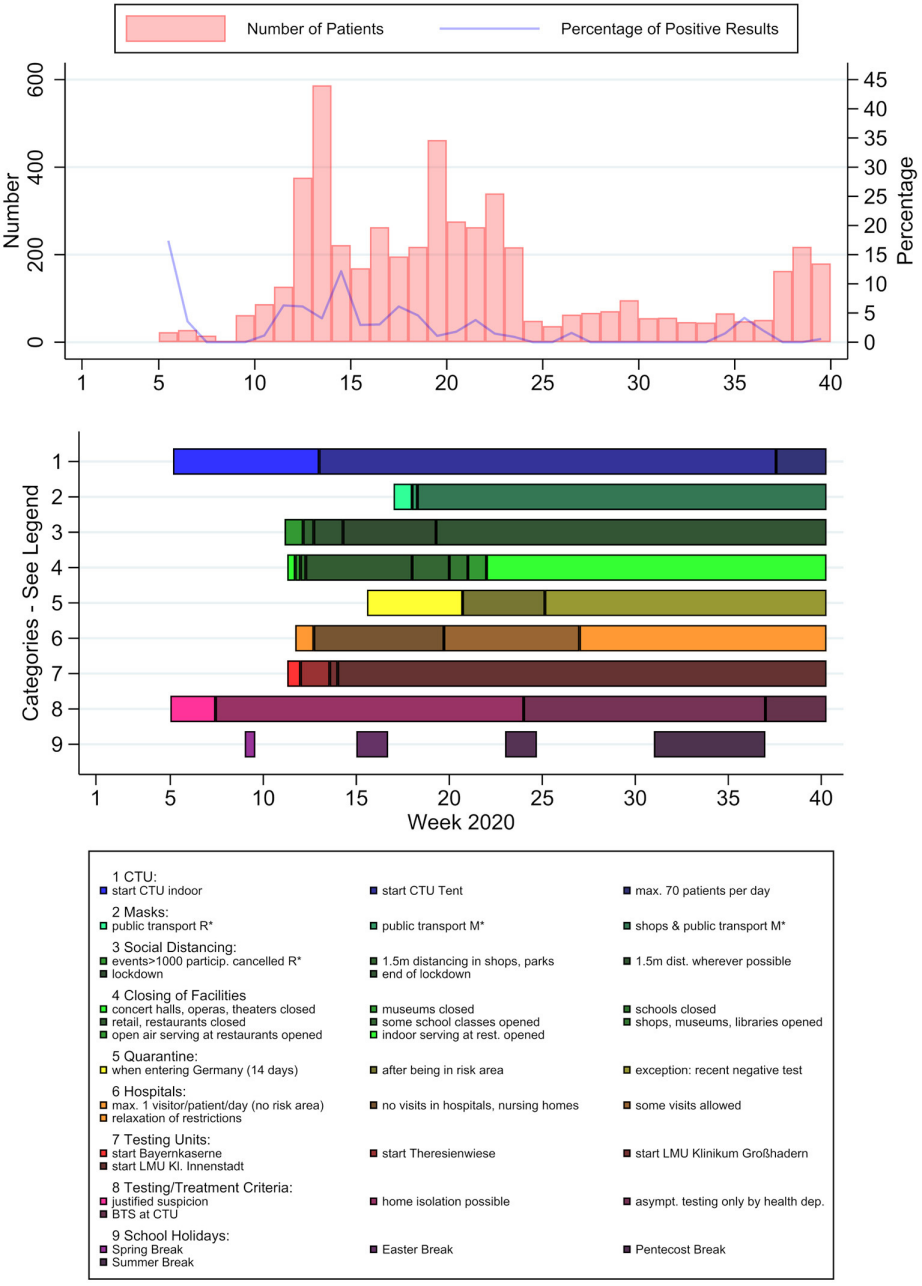
PCR-test results and potential influencing factors over time. Patient numbers (light red) and positive rates (blue) per calendar week, timeline of potential influencing factors. *R, recommendation; *M, mandatory.

Possible influencing factors in patient numbers and characteristics detected through literature research and key informant interviews could be divided into several categories (Figure 5): Internal influences on capacity included the set-up of a testing-specific outdoor tent with an assembly-line principle in week 12–13, and an admittance of maximum 70 patients per day due to limited workforce from week 37 to 40. Regarding public health and safety measures, social distancing measures, the closing of facilities and visitor restrictions in hospitals and nursing homes can be observed to have been implemented from week 11 to 12 on, whereas regulations concerning the wearing of face masks were implemented relatively late and were only mandatory from week 18 on. Category 7 shows the initiation of other testing facilities, which were put into operation between week 11 and 14. The most crucial changes in testing and treatment policies included the decision to home-isolate positive patients with mild symptoms instead of hospitalization (week 7), a notice by the German federal ministry of health about asymptomatic testing only by specific authorized institutions (published in week 24) and the implementation of the Bavarian testing strategy (BTS) in week 37. Additionally, national school holidays can be seen in Figure 5.

## Discussion

Our data can be seen as a depiction of the interaction between the real course of the pandemic and a multitude of public measures. The general form of our curve largely coincides with the state-wide course of positive test results during the first and the beginning of the second wave in Bavaria (24). The substantial trend changes in patient numbers and characteristics can nevertheless most fittingly be described by testing capacities, policy changes and individual risk and health seeking behavior: The close to exponential growth until week 13 (as seen in the breakpoint model) was capacitated through the implementation of home isolation instead of facility in-house monitoring of low-risk patients and the increase of spatial capacity. The initiation of other testing units entailed a drop in patient numbers, most vividly after week 13 with the testing units at the university hospitals Klinikum Großhadern and Innenstadt in week 13–14. A drop after week 24 correlates with asymptomatic testing only to be conducted by pre-specified institutions. On the contrary, the implementation of the BTS with indication-free testing saw a rise in patients from the general population (group “Others”) with light or no symptoms.

The decrease in median days since symptom onset at presentation after April could be contributed to previously constrained mobility due to the situation of a general lockdown and later on a raising awareness about COVID-specific symptoms in the public or better availability of tests.

During spring and summer break, a rise in returning travelers could be observed, whereas during Easter and Pentecost, with lockdown and stricter travel restrictions, there was no notable increase in reported travels abroad. Most presentations of travelers after spring break can be attributed to skiing resorts in Austria and Italy, which have previously been described as grounds of major spreading events (25). In the period of the summer holidays, the most common destinations reported were in the Mediterranean region, being explainable by the significant number of healthcare workers in Germany with a Balkan family background.

### Limitations

Several limitations to this study need to be acknowledged: With relatively small patient numbers and a specific cohort with significant pre-test selection, our presented results carry limited external validity. Our statistical model was, unlike other epidemiological models that allow for prospective predictions (4, 5), exclusively intended for retrospective analysis and is therefore only suitable for predictive forecasting to a limited extent. Additionally, the intermittent triage for symptomatic patients may have led to reporting of non-existent symptoms. Through changing CRF questions over time, information about the exposition of patients in the early phase of the pandemic could not be included. However, the changing patient composition as well as the evolution of case reporting criteria may be considered part the nature of the testing unit and hence one assertive statement of this report. Language barriers, as well as a multitude of social influencing factors, could possibly be responsible for underreporting of exposures and symptoms.

## Conclusions

Considering the beforementioned limitations, our findings might not be consistent with the results of other organizations tasked with testing for SARS-CoV-2. Nevertheless, we believe our observations to demonstrate the volatility of patient numbers and characteristics in correlation with multivarious influencing factors, whose individual effects remain challenging to distinguish. Additionally, previous findings suggest only a fraction of infections to have been detected through conventional testing (26, 27). Furthermore, differences in triage might limit the applicability of the incidence as a comparative measure between different regions or countries. Thus, we believe the incidence to not be the best suitable parameter to indicate the infection rate in the general public or to base public health and safety measures on. Free of charge, low threshold testing such as the Bayerische Teststrategie, although costly and still prone to errors, might facilitate to increase the validity of the incidence as a measurement for the infection rate in a population. Other suitable indicators include the rate of intensive care unit admissions, sentinel tests (repeated testing of specific subpopulations) and representative samples.

In an early pandemic with a rather unfamiliar pathogen knowledge, policies, and relevant patient groups are rapidly changing. Organizations tasked with testing should expect and prepare for significant fluctuations in patient numbers. Many independent factors, such as public health measures or social media spread of public opinions may eventually result in relevant changes in patient behavior. For these reasons, a flexible and quickly adaptable structure is urgently needed for testing. Wherever feasible, a transfer of knowledge to cooperating institutions working with vulnerable groups should be aimed for to build resources and to strengthen the public health response.

## Data Availability Statement

The raw data supporting the conclusions of this article will be made available by the authors, without undue reservation.

## Ethics Statement

The studies involving human participants were reviewed and approved by Ethikkommission bei der Medizinischen Fakultät der LMU München. Written informed consent from the participants' legal guardian/next of kin was not required to participate in this study in accordance with the national legislation and the institutional requirements.

## Author Contributions

CH, GF, and HH conceived the study and conducted the data analysis. GF and HH wrote the manuscript. CJ, CR, and MH participated in the qualitative data analysis. All authors read and approved the final version of the manuscript.

## Funding

HH has received a scholarship by LMU. The research work and compilation of the manuscript has not received any third-party funding.

## Conflict of Interest

The authors declare that the research was conducted in the absence of any commercial or financial relationships that could be construed as a potential conflict of interest.

## Publisher's Note

All claims expressed in this article are solely those of the authors and do not necessarily represent those of their affiliated organizations, or those of the publisher, the editors and the reviewers. Any product that may be evaluated in this article, or claim that may be made by its manufacturer, is not guaranteed or endorsed by the publisher.
