# Supplementary material for: COVID-19 Testing Unit Munich: Impact of Public Health and Safety Measures on Patient Characteristics and Test Results, January to September 2020
Source: Front Public Health. 2022 Mar 22;10:856189. doi: 10.3389/fpubh.2022.856189 (PMC8980425; doi:10.3389/fpubh.2022.856189)
Supplement: Supplementary file 1 [file Data_Sheet_1.PDF]

Supplementary file 1. CTU Case Report Form.

COVID-19 Testing Unit - Registration Form

1. Personal data

Name	first name	Gender ☐ f ☐ m
Date of birth	Cell phone number (please write legibly!)	Health insurance ☐ statutory ☐ private
Street, house number	POS-	Place of residence

2. Professional data

Employer	Workplace (e.g. ward/ KUM staff: campus
Professional group	

3. Exposure (within the last 14 days)

Close contact to confirmed case<br>☐ Employees ☐ Patient / Client ☐ private environm ☐ other ☐ not applicable	
Other exposure (e.g. stay in designated COVID-19 risk area)	
Earliest possible date of exposure (if known)	Latest possible date of exposure (if known)

4. Current symptoms

Are there any current<br>☐ no ☐ yes, since (date):
if yes: What are the current symptoms?<br>☐ Fever ☐ Cough ☐ Shortness of breath ☐ Sore throat<br>☐ Runny nose ☐ loss of sense of smell and/or taste<br>☐ Headache ☐ others: ________________________________

5. Vital parameters (to be taken and filled in CTU staff)

Temperature________________	Oxygen saturation:__________	Pulse: _____

6. Results release

The result of the test carried out will be communicated to the occupational health service responsible for you (if applicable). In addition, each patient receives his or her result promptly by SMS (please check your mobile phone number!).

☐ * I hereby confirm that I have read the "Patient information on data protection". I agree to the "Treatment Contract for Outpatient Treatment", the "Declaration of Consent on Data Protection" and the "Contract on the Use of Outpatient Private Medical Services" (the latter only for private patients) in the form available as a pdf document on the homepage of the Tropical Institute Munich (all documents are available at www.tropinst.med.uni-muenchen.de).

☐ * I am aware that the billing of the diagnostics is done by my statutory or private health insurance company. The Tropical Institute explicitly does not clarify whether this is a competence of the professional association.

I agree to be contacted by phone for SARS-CoV-2 specific study purposes if necessary:
☐ yes ☐ no

Date: ___________Signature (in case of minors, signature of legal guardian) __________________________

INTERNAL use (please DO NOT fill in):

Test result:	positive ☐	negative ☐	Result notification:	Patient ☐
Initial testing:	yes ☐	no ☐		Health Authority ☐
if no, when: first test done on ___.___.______				Occupational health ☐

☐ Follow-up test no. ___

☐ Testing after re-exposure
